# Epigenetic and genetic deregulation in cancer target distinct signaling pathway domains

**DOI:** 10.1093/nar/gkw1100

**Published:** 2016-11-28

**Authors:** Yang Gao, Andrew E. Teschendorff

**Affiliations:** 1 CAS Key Lab for Computational Biology, CAS-MPG Partner Institute for Computational Biology, Chinese Academy of Sciences, Shanghai Institute for Biological Sciences, 320 Yue Yang Road, Shanghai 200031, China; 2 University of Chinese Academy of Sciences, No.19A Yuquan Road, Beijing 100049, China; 3 Department of Women's Cancer, University College London, 74 Huntley Street, London WC1E 6BT, UK; 4 Statistical Genomics Group, Paul O'Gorman Building, UCL Cancer Institute, University College London, 72 Huntley Street, London WC1E 6BT, UK

## Abstract

Cancer is characterized by both genetic and epigenetic alterations. While cancer driver mutations and copy-number alterations have been studied at a systems-level, relatively little is known about the systems-level patterns exhibited by their epigenetic counterparts. Here we perform a pan-cancer wide systems-level analysis, mapping candidate cancer-driver DNA methylation (DNAm) alterations onto a human interactome. We demonstrate that functional DNAm alterations in cancer tend to map to nodes of lower connectivity and inter-connectivity, compared to the corresponding alterations at the genomic level. We find that epigenetic alterations are relatively over-represented in extracellular and transmembrane signaling domains, whereas cancer genes undergoing amplification or deletion tend to be enriched within the intracellular domain. A pan-cancer wide meta-analysis identifies WNT and chemokine signaling, as two key pathways where epigenetic deregulation preferentially targets extracellular components. We further pinpoint specific chemokine ligands/receptors whose epigenetic deregulation associates with key epigenetic enzymes, representing potential targets for epigenetic therapy. Our results suggest that epigenetic deregulation in cancer not only targets tissue-specific transcription factors, but also modulates signaling within the extra-cellular domain, providing novel system-level insight into the potential distinctive role of genetic and epigenetic alterations in cancer.

## INTRODUCTION

Cancer is a complex disease that is caused by genetic and epigenetic alterations (1). Somatic mutations, copy-number and DNA methylation (DNAm) aberrations are all known to influence transcript abundance in tumors, and thus may define different types of ‘driver’ events (2–6). Many studies have mapped somatic mutations and copy-number aberrations onto signaling pathways and human protein interactomes, revealing a number of ‘system-level’ insights (7–14). For instance, such events map to central positions in protein–protein interaction (PPI) networks (7,11,12) and often exhibit patterns of mutual exclusivity within signaling pathways (15,16).

In contrast to genomic alterations, it is only more recently that studies have begun to explore how cancer-related DNAm aberrations map onto signaling pathways and protein interactomes. For instance, some previous studies have shown that cancer-associated DNAm changes tend to cluster in such PPI networks, allowing interactome hotspots of differential DNAm or of simultaneous differential DNAm and mRNA expression, to be identified (6,17,18). In the context of aging, it has been found that age-associated DNAm drift occurs preferentially at genes of exceptionally low connectivity that occupy peripheral network positions, in stark contrast to other age-related genes, including longevity- and disease-associated genes (19). A similar pattern was observed by Cheng (20) in the context of differentially methylated genes associated with cancer survival. However, no study has yet conducted an in-depth comparison of the systems-level properties of epigenetic versus genetic alterations in cancer.

The recent TCGA pan-cancer resource (21) now allows for such an in-depth comparison. Specifically, we decided to conduct a pan-cancer wide analysis at a systems-level, using a highly curated PPI network, in order to address the following unexplored questions. First, do network topological properties of functional DNAm aberrations in cancer differ from those of functional somatic copy-number alterations (SCNA) or those of cancer driver mutations? Second, do epigenetic and genetic driver alterations target different domains within the cell's signaling hierarchy? Third, are there any particular signaling pathways that are more prone to epigenetic versus genetic disruption, and if so, does this depend on the signaling domain of the pathway?

Our pan-cancer analysis reveals that genetic and epigenetic alterations not only target genes with different topological properties, but that these genes also exhibit a differential pattern in terms of the cell's signaling domain architecture. These novel systems-level insights are consistent with the view that a proportion of epigenetic cancer driver events are mediated by extrinsic factors, i.e. the cellular environment.

## MATERIALS AND METHODS

### Datasets

#### Gene expression data

We downloaded the RNA-SeqV2 level 3 expression data from TCGA (December 2014) for ten cancer types, including breast invasive carcinoma (BRCA) (22), bladder cancer (BLCA) (23), colon adenocarcinoma (COAD) (24), head and neck squamous cell carcinoma (HNSC) (25), kidney renal carcinoma (KIRC) (26), liver hepatocellular carcinoma (LIHC) (27), lung adenocarcinoma (LUAD) (28), lung squamous cell carcinoma (LUSC) (29), thyroid carcinoma (THCA) (30) and uterine corpus endometrial carcinoma (UCEC) (31) (The sample IDs are provided in Supplementary Table S1). The level 3 RNA-Seq data were processed further as follows: (i) zeroes were substituted by the minimum positive value of the dataset; (ii) expression values were then log2 transformed in order to regularize the data. (iii) Inter-array normalization was then performed using the limma package (32).

#### DNA methylation data

For the 10 cancer types mentioned above, DNAm data generated with the Illumina Infinium HumanMethylation450 BeadChip array were downloaded from TCGA data portal. Probes with missing data (i.e. NAs) in more than 30% of the samples were removed. The rest of the probes with NAs were imputed using the k-nearest neighbors (knn) (k=5) imputation procedure (33). Subsequently, BMIQ was used to correct for the type II probe bias (34).

#### Somatic copy number data

For the 10 cancer types mentioned above, we downloaded TCGA level-3 copy number segmentation data, which were generated from the Affymetrix SNP 6.0 platform. We selected those files with probes sorted according to the hg19 reference genome, and with probes mapping to germline CNVs removed prior to segmentation. When calling alterations, thresholds were set based on the median of the log2 ratio for each array +2σ or −2σ computed using top 50% of the probes (ordered by their log ratios) for calling gains and losses, respectively. The median of the log2 ratio +4σ or −4σ was used to call amplification and deletion respectively. In order to identify the genes and features that were altered by copy number aberrations, we searched for overlap of segments with gene regions. The complete gene coordinates was given by hg19 using R package TxDb.Hsapiens.UCSC.hg19.knownGene. A patient-by-gene call matrix was generated following a similar procedure to (2) to capture different perspectives of gene alterations, which has values representing discrete copy number states. For each patient p and each gene g, we identified segments s that overlap g and assign C(p, g) with the copy number state of s. If gene g overlaps or is broken by a set of segments, S = s1, …, sk, where k ≥2, the copy number state of the segment with maximal severity(‘Neutral’ < ‘Gain/Loss’ < ‘Amplification/Deletion’) was assigned, where ties were broken in samples exhibiting both a loss and gain according to the maximal absolute value of the segmented mean. The density distribution of non-zero entries of copy number call matrices across 10 cancer types are shown in Supplementary Figure S1.

#### Mutation data

For the ten cancer types mentioned above, all mutation annotation format files were downloaded from the TCGA.

### Defining differentially methylated and differentially expressed genes

To assign DNAm values to a given gene, we assign to a gene the average value of probes mapping to within 200 bp of the transcription start site (TSS) of this gene. If no probes map to within 200 bp of the TSS, we use the average of probes mapping to the first exon of the gene. If such probes are also not present, we use the average of probes mapping to within 1500 bp of the TSS. Justification for this procedure is provided in (18). Probes mapping to the gene body are not used. Using this gene-based methylation value, we then compute moderated t-statistics using an empirical Bayes framework (32). The same empirical Bayes procedure was applied to gene expression data. Methylation differences with false discovery rate (FDR) <0.05 and with absolute difference in mean methylation beta levels between the two groups of more than 0.1 were considered statistically significant. Gene expression differences with FDR <0.05 and with a log2 fold change between two groups of more than 1 were considered statistically significant. Using both t-statistics for each gene, we then selected genes with opposite signs of t-statistics, which indicates an anti-correlation between DNAm and mRNAm expression, and further divided them into two groups based on the directionality of differential methylation: a hypermethylated group (HyperM) and a hypomethylated (HypoM) group. Genes in each group were ranked according to the integrative statistic, as described in (18). Finally, we further filtered the gene lists using a multivariate regression framework of gene expression against DNAm and CNV as covariates (and using both normal and cancer samples), to select genes exhibiting a significant anti-correlation between mRNA expression and DNAm. This was done to ensure that (i) the anti-correlation between differential mRNA expression and differential DNAm is due to the same set of tumours, and (ii) to ensure that the anti-correlation between DNAm and mRNA expression cannot be explained by concomitant alterations at the CNV level.

### Finding somatic copy number altered genes

After deriving the copy number call matrix, as described above, we developed a procedure to identify those SCNA that are associated with a corresponding change in gene expression. Gaussian distributions were fitted to the log2 expression values for each gene and for each cancer type, using maximum likelihood estimates of the mean and variance. Based on this distribution, we can derive a simplified vector for each gene, where samples with expression in the 5% left tail were marked as underexpressed and samples with expression in the 5% right tail were marked as overexpressed. Therefore, for each gene in each sample, we have information as to whether it defines an amplification/deletion and overexpression/underexpression event. From this, we generate two binary matrices: one is an Amplification/Overexpression call matrix where a matrix entry is assigned 1 if it is both an amplification and an overexpression event, and 0 otherwise. The other matrix is a Deletion/Underexpression call matrix where a matrix entry is assigned 1 if it is both a deletion and an underexpression event, and 0 otherwise. For each call matrix, we select genes that have at least one non-zero entry and then rank the genes based on the number of non-zero entries in decreasing order. The resulting two ordered gene lists correspond to SCN gained and overexpressed genes (Amplification) and SCN deleted and underexpressed genes (Deletion). Finally, we further filtered the gene lists using a multivariate regression framework of gene expression against DNAm and CNV as covariates (and using both normal and cancer samples), to select genes exhibiting a significant correlation between mRNA expression and CNV. This was done to ensure that (i) the correlation between differential mRNA expression and SCNA is due to the same set of tumours, and (ii) to ensure that the correlation between SCNA and mRNA expression cannot be explained by concomitant alterations at the DNAm level.

### Finding significantly mutated genes

We use the MutSigCV software (35) to identify significantly mutated genes which takes DNA replication time, expression and chromatin state into account when estimating the background mutation rate.

### Finding epigenetically regulated tissue-specific genes

We generated the epigenetically regulated tissue-specific gene lists for each of 10 tissue types by comparing the DNAm data as well as gene expression data of normal samples from one tissue type with that of other nine tissue types using an empirical Bayes framework (32). Methylation differences with FDR <0.05 and at least 30% of mean methylation difference between two groups were considered statistically significant. Gene expression differences with FDR <0.05 and log2 fold change between two groups more than 2 were considered statistically significant. Using both t-statistics for each gene, we then selected genes with opposite signs of t-statistics, which indicates an anti-correlation between DNAm and mRNA, and further divided them into two groups based on the directionality of differential methylation: a hypermethylated group (HyperM) and a hypomethylated group (HypoM). Genes in each group were ranked according to the integrative statistic, as described in (18). The significance of overlap between epigenetically regulated tissue-specific gene lists and epigenetically regulated cancer altered gene lists for each cancer type was evaluated using one tailed Fisher's exact test.

### Protein interaction network (PIN)

We used the 2015 March version from the Pathway Commons (PC2) database (36) to build the PIN. In detail, this was built by integrating the Human Protein Interaction Database (HPRD), the National Cancer Institute Nature Pathway Interaction Database (NCI-PID), the Interactome (Interact) and the Biological General Repository for interaction Datasets (BioGRID). Protein interactions included stable interactions like those defining protein complexes as well as transient interactions like post-translational modifications and enzymatic reactions found in signal transduction pathways. We focused on the largest connected component of genes with Entrez ID identifiers, which amounted to a connected network of 15 728 nodes and 1 910 396 interactions. This PIN was further pruned by removing edges which were not consistent with the signaling domain hierarchy structure (see below for definition of signaling domains). Thus, only edges with corresponding end nodes in the following signaling domain combinations were allowed: EC-EC, EC-MR, MR-IC and IC-IC, where EC = extra-cellular, IC = intra-cellular, MR = membrane-receptor. This resulted in a reduced PIN of 10 726 nodes and 1 306 162 interactions (maximally connected component). The sparsity (i.e. the fraction of edges to total number of possible edges) of this PIN is 0.023.

### Definition of signaling domains

Following (37), we annotated genes into five distinct signaling domains: growth modulators (GM), secreted factors (SF), membrane receptors (MR), intracellular receptor substrates (ICRS) and intracellular non receptor substrates (ICNRS). These assignments were made using main cellular localization data of the corresponding proteins, as given in the HPRD database. Specifically, we first defined an intra-cellular domain as all those GO-terms containing the following terms: ‘Nucleus’,‘Cytoplasm’,‘Ribosome’,‘Nucleolus’,‘Mitochondri’,‘Endoplasmic reticulum’,‘Golgi’,‘Lysosome’,‘Cytosol’,‘Cytoskeleton’,‘Nuclear’,‘Kinetochore’,‘Chromosome’,‘Endosome’,‘Intracellular’,‘Nucleoplasm’,‘Perinuclear’,‘Centrosome’,‘Peroxisome’, ‘Microtubule’,‘Microsome’,‘endosome’,‘Centriole’,‘Sarcoplasm’,‘Secretory granule’,‘Endocytic vesicle’,‘cytoskeleton’,‘Peroxisomal membrane’,‘Acrosome’,‘Zymogen granule’. For the membrane-receptor (MR) domain we used: ‘Plasma membrane’,‘Integral to membrane’,‘Cell surface’,‘Integral to plasma membrane’,‘Cell projection’,‘Basolateral membrane’,‘Axoneme’,‘Apical membrane’ and for the extra-cellular (EC) domain:

‘Extracellular’,‘Cell junction’,‘Synapse’,‘Dendrite’,‘Secreted’,‘Synaptic vesicle’. The IC class was subdivided into ICRS and ICNRS subclasses, according to whether the IC annotated protein interacts with a MR (if yes, then ICRS) or not (ICNRS). Similarly, the EC class was subdivided further into GM and SF subclasses, according to whether the EC annotated protein interacts with a MR (SF) or not (GM).

Because genes may be annotated to multiple signaling domains, for some analysis we used a coarse grained 2-domain assignment, whereby a gene annotated to both extracellular and transmembrane domains was allocated to ‘EC’, and a gene annotated to both transmembrane and intracellular domains was allocated as ‘IC’.

### Comparison of shortest path distances among genes in the different alteration groups

To assess the inter-connectivity of the group of genes in each alteration group in the PIN, we compared the distribution of the shortest path length between every pair of genes within an alteration group and within each cancer type. We selected the top 100 ranked genes with different alteration types, including (i) Hypermethylated and underexpressed genes (HyperM), (ii) Hypomethylated and overexpressed genes (HypoM), (iii) SCN gained and overexpressed genes (Amplification), (iv) SCN deleted and underexpressed genes (Deletion), (v) Mutated genes (Mutation). The shortest paths length was estimated for each gene pair in the top-ranked list, and the average shortest path lengths were compared between different alteration groups within one cancer type. The comparison was done by computing the *P*-values using one-tailed paired Wilcoxon rank-sum test between any two different alteration types and for each of the 10 cancer types separately. For HyperM and other four groups, we tested whether HyperM group has significantly larger average shortest paths length than the other four groups. For HypoM and other three groups (except HyperM), we tested whether HypoM group has significantly larger average shortest paths length than the other three alteration groups. For Mutation and Amplification/Deletion, we tested whether Mutation group has significantly larger average shortest paths length than Amplification/Deletion groups. For Amplification and Deletion group, we tested whether Amplification group has significantly larger average shortest paths length than Deletion group.

### Comparison of signaling domain distribution within PIN

We did the enrichment analysis of signaling domains for each alteration group by comparing the number of observed genes in each domain with the number of expected genes in each domain. This expected number is the number of genes in an alteration group multiplied with the percentage of genes in each signaling domain. Here we combine the extracellular and transmembrane domain as one big domain (EC+MR) (see subsection on signaling domain definitions), and use intracellular domain as the other domain. The odds ratio (OR) and *P*-values were calculated using the one-tailed Fisher's exact test. These analyses were done in two different ways: (i) using all the significant genes in each alteration group, and (ii) selecting the same number of top-ranked genes for each alteration group, which was chosen as the minimum number for all five groups as determined in (i).

### Comparison of signaling domains distributions in specific pathway

We downloaded signaling pathway information from MSigDB (38). For each signaling pathway, we calculated the number of genes undergoing functional DNAm or SCN alterations and which mapped into either the extracellular (EC) or intracellular (IC) domain. A *P*-value was computed using a one-tailed Fisher's exact test to determine whether genes with functional DNAm aberrations were enriched in the EC domain compared with SCNAs. A meta-analysis *P*-value was computed by Fisher's combined test for each signaling pathway across 10 cancer types, and signaling pathways with meta-analysis *P*-values below than 0.05 were deemed to exhibit a significant differential signaling domain distribution between functional DNAm and SCN alterations. To identify signaling pathways that exhibit functional DNAm alterations preferentially in the extracellular domain, we calculated the number of genes undergoing functional DNAm alterations in the extracellular and intracellular domains, respectively, and compared them to the numbers of genes in these domains not exhibiting functional DNAm alterations. *P*-value was computed using a one-tailed Fisher's exact test and a meta-analysis *P*-value was computed by using Fisher's combined test for each signaling pathway across 10 cancer types.

## RESULTS

### Construction of putative DNAm and SCNA driven cancer gene lists

We downloaded TCGA Illumina 450k DNAm, SCNA and RNA-Seq gene expression data for a total of 10 cancer types for which there were reasonable numbers of normal samples (‘Materials and Methods’ section, Supplementary Table S1). We asked if functional DNAm alterations in cancer are distinguishable from functional SCNAs in the context of how they map onto a highly curated PPI network (‘Materials and Methods’ section). This analysis was performed by considering four separate classes of putative driver cancer genes: (i) genes which exhibit a hypermethylated promoter and underexpression in cancer, (ii) genes which exhibit a hypomethylated promoter and overexpression in cancer, (iii) genes with SCN loss and underexpression in cancer and (iv) genes with SCN gain and overexpression in cancer. The identification of these putative driver cancer gene sets used state-of-the-art methods, which have previously been used to successfully identify known driver genes at both SCN and DNAm levels (‘Materials and Methods’ section) (6,18,39). For instance, we applied the method used in the breast cancer METABRIC study of Curtis *et al.* (39) to identify SCN cancer drivers in the TCGA breast cancer set, revealing a highly significant overlap of the TCGA-derived driver list with the one derived from METABRIC (Supplementary Figure S2). In the case of DNAm, we ignored genes where the promoter DNAm change was not anti-correlated to gene-expression change, since positive correlations represent the minority of associations (18) and are less likely to be linked causally (40). We note that this approach of focusing on anti-correlated patterns between promoter DNAm and gene expression was used by us previously, successfully identifying a causal driver of endometrial cancer, the causal association of which was validated experimentally (6,18). Besides imposing stringent levels of statistical significance, we also demanded that differences in DNAm and mRNA expression between normal and cancer be at least 10% and larger than 2-fold, respectively (Materials and Methods). Since DNAm and SCN variation can simultaneously affect gene expression, our selection procedure further filtered genes according to statistical significance in multivariate regression models with mRNA expression as the response variable and including DNAm and SCNA as predictors (‘Materials and Methods’ section). The resulting sets and numbers of genes for each of the 4 putative cancer driver classes in each of the 10 TCGA cancer types are listed in Supplementary Tables S2–5. The range in the number of genes in each class across cancer types was (16 642), (94 368), (205 1877) and (161 1242) for HyperM, HypoM, Amplification and Deletion respectively. We note that for a given TCGA cancer type, the overlap between these 4 gene lists was minimal, especially between the DNAm and SCNA groups (Supplementary Figure S3).

### Functional DNA methylation alterations in cancer exhibit lower interactome connectivity compared to corresponding SCNAs and mutations

**Figure 1.**
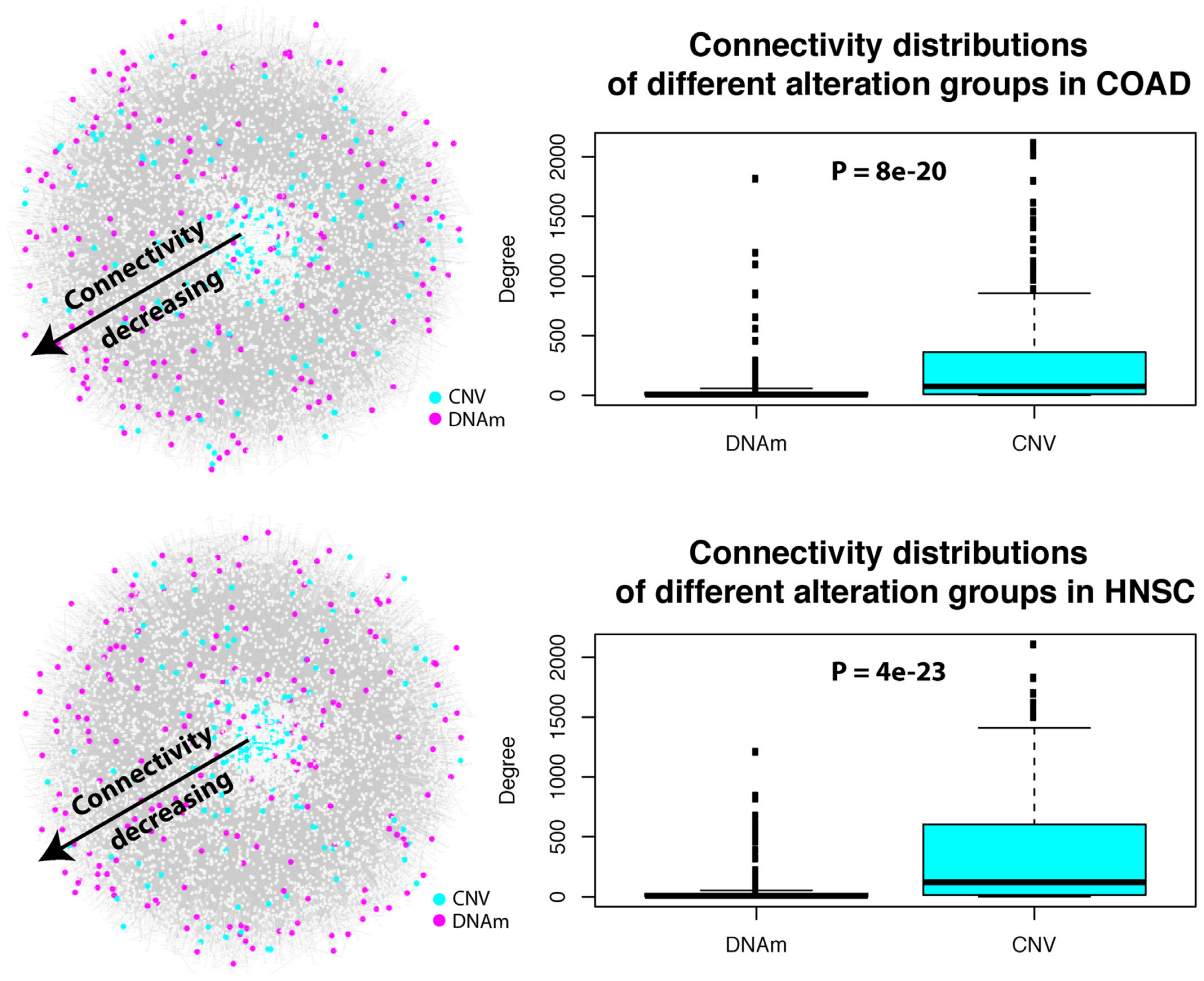
Functional epigenetic alterations exhibit lower interactome connectivity than their SCNA counterparts. Left panels: for two cancer types (COAD and HNSC), the PPI network is depicted (interactions have been suppressed) with nodes (genes/proteins) colored according to the type of functional alteration and with the radial distance from the center indicating their connectivity (nodes in the center have higher connectivity and connectivity decreases radially outward). We defined for each cancer type four types of functional alterations at the gene-level, including the 100 top-ranked (i) hypermethylated and underexpressed genes (HyperM), (ii) hypomethylated and overexpressed genes (HypoM), (iii) gain of copy-number and overexpressed genes (Amplification) and (iv) CN deleted and underexpressed genes (Deletion). Classes (i)+(ii) are shown here as one group (DNAm) indicated by color magenta, while classes (iii)+(iv) represent another group (CNV) indicated by color cyan. **Right panels**: Boxplots of the connectivity (degree) for the same two groups of genes. The *P*-value is from a Wilcoxon rank sum-test comparing the connectivity of genes exhibiting simultaneous differential methylation and differential expression (i.e. classes (i)+(ii)) versus the connectivity of genes exhibiting simultaneous CNV and differential expression (classes (iii)+(iv)). Analogous plots for all other cancer-types are shown in Supplemental Figure S4.

In order to objectively compare the topological properties of these different putative cancer driver gene classes in a PPI network, we need to select a given identical number of top-ranked genes from each class. For a given TCGA cancer type, we thus mapped the top-100 ranked genes from each class onto our PPI network of 10 726 nodes and 1 306 162 edges (‘Materials and Methods’ section). For each set of top ranked genes, we studied the distribution of their connectivities/degrees (i.e. the number of nearest neighbors of each gene in the list) in the network. In each of the 10 cancer-types we observed a highly statistically significant difference (Wilcoxon rank sum test *P* < 1e-5), with genes undergoing differential methylation and differential expression exhibiting a significantly lower connectivity compared to genes undergoing simultaneous SCN and gene expression alterations (Figure 1; Supplementary Figures S4 and 5). We also observed statistically significant differences between the hypermethylated/underexpressed gene class and the hypomethylated/overexpressed class in five cancer types, with the former exhibiting lower connectivity (Table 1).

**Table 1.** Putative functional epigenetic drivers exhibit lower connectivity than their functional SCN counterparts

*P*-values of one-tailed Wilcoxon test comparing connectivity					
COAD		HyperM	HypoM	Amplification	Deletion
	HyperM	NA	0.95	1	1
	HypoM	0.05	NA	1	1
	Amplification	6e-13	5e-07	NA	0.921
	Deletion	1e-14	1e-08	0.079	NA
HNSC		HyperM	HypoM	Amplification	Deletion
	HyperM	NA	0.991	1	1
	HypoM	0.009	NA	1	1
	Amplification	7e-14	1e-09	NA	0.578
	Deletion	3e-15	2e-11	0.423	NA
BLCA		HyperM	HypoM	Amplification	Deletion
	HyperM	NA	0.97	1	1
	HypoM	0.03	NA	1	1
	Amplification	4e-12	2e-07	NA	0.153
	Deletion	4e-08	1e-04	0.848	NA
BRCA		HyperM	HypoM	Amplification	Deletion
	HyperM	NA	0.729	1	1
	HypoM	0.272	NA	1	1
	Amplification	1e-10	4e-09	NA	0.034
	Deletion	2e-05	2e-04	0.966	NA
KIRC		HyperM	HypoM	Amplification	Deletion
	HyperM	NA	0.734	1	1
	HypoM	0.266	NA	1	1
	Amplification	6e-06	4e-05	NA	0.999
	Deletion	6e-14	1e-12	9e-04	NA
LIHC		HyperM	HypoM	Amplification	Deletion
	HyperM	NA	0.65	1	1
	HypoM	0.351	NA	1	1
	Amplification	4e-13	3e-09	NA	0.51
	Deletion	1e-10	8e-08	0.491	NA
LUSC		HyperM	HypoM	Amplification	Deletion
	HyperM	NA	1	1	1
	HypoM	3e-04	NA	1	0.991
	Amplification	2e-12	1e-04	NA	0.035
	Deletion	5e-10	0.009	0.965	NA
LUAD		HyperM	HypoM	Amplification	Deletion
	HyperM	NA	0.247	1	1
	HypoM	0.754	NA	1	1
	Amplification	8e-14	2e-13	NA	0.294
	Deletion	3e-10	2e-10	0.707	NA
THCA		HyperM	HypoM	Amplification	Deletion
	HyperM	NA	0.254	0.849	0.938
	HypoM	0.757	NA	1	1
	Amplification	0.155	3e-04	NA	0.98
	Deletion	0.064	1e-06	0.02	NA
UCEC		HyperM	HypoM	Amplification	Deletion
	HyperM	NA	0.998	1	1
	HypoM	0.002	NA	1	1
	Amplification	6e-12	6e-06	NA	0.611
	Deletion	4e-14	5e-07	0.39	NA

Table lists one-tailed Wilcoxon rank sum test *P*-values comparing the connectivity (degree) distribution of the top ranked 100 genes in each molecular alteration group between each other, and for each of 10 TCGA cancer types. For each cancer type, the alternative hypothesis being tested is that the connectivity of the gene-class in the column is smaller than that of the gene class indicated in the row.

Next, we obtained the distribution of the shortest path length between all gene-pairs within one of the four classes and asked if their distributions differed. This assesses how close the corresponding genes in each set are to each other in the network. We also included the top 100 ranked genes based on mutational frequency (‘Materials and Methods’ section, Supplementary Table S6). This analysis showed that, across all 10 cancer-types, genes undergoing differential methylation and differential expression generally exhibited longer shortest path lengths, compared to genes undergoing simultaneous SCN and gene expression alterations, or to frequently mutated genes (Supplementary Figure S6 and Supplementary Table S7). We note that the longer shortest path lengths exhibited by genes undergoing differential methylation and differential expression is consistent with their lower node-connectivity.

### Functional epigenetic and genetic cancer alterations map preferentially into different signaling pathway domains

**Figure 2.**
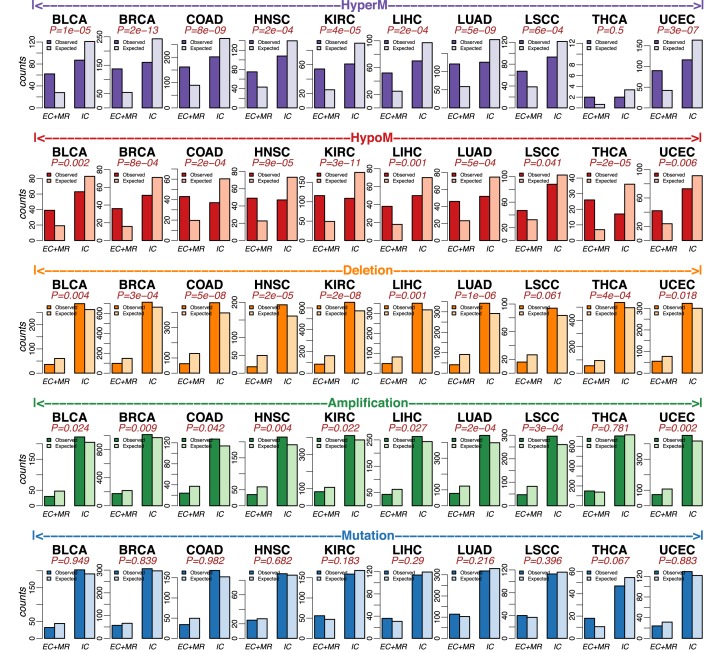
Functional epigenetic alterations preferentially target genes in the extra-cellular/transmembrane domains, with SCNA counterparts preferentially mapping to the intra-cellular domain. **Top Row:** Barplots comparing the observed and expected numbers of genes for two different signaling domains (extracellular+transmembrane receptor: EC+MR, and intra-cellular: IC) for HyperM group (hypermethylated and underexpressed genes) across 10 TCGA cancer types. *P*-values are from a one-tailed Fisher's exact test. Alternative hypothesis is that the odds ratio of finding more genes mapping to the EC+MR domain is >1. **Row-2:** As before but for the HypoM group (hypomethylated and overexpressed genes). *P*-values are from a one-tailed Fisher's exact test. Alternative hypothesis is that the odds ratio of finding more genes mapping to the EC+MR domain is >1. **Middle Row:** As before but for the Deletion group (SCN deletion and underexpressed genes). *P*-values are from a one-tailed Fisher's exact test. Alternative hypothesis is that the odds ratio of finding more genes mapping to the EC+MR domain is <1. **Second last row:** As before but for Amplification group (SCN gain and overexpressed genes). *P*-values are from a one-tailed Fisher's exact test. Alternative hypothesis is that the odds ratio of finding more genes mapping to the EC+MR domain is <1. **Last row:** As before, but for Mutation group (mutated genes). *P*-values are from a one-tailed Fisher's exact test. Alternative hypothesis is that the odds ratio of finding more genes mapping to the EC+MR domain is >1.

Important properties such as gene expression variance are known to vary according to the gene's signaling domain (37). Following Komurov (37), we henceforth categorized all genes of our PPI network into five signalling hierarchy classes: (i) growth modulators (GM), (ii) secreted factors (SF), (iii) membrane receptors (MR), (iv) intracellular receptor substrates (ICRS) and (v) intracellular non-receptor substrates (ICNRS) (‘Materials and Methods’ section). We validated our signaling domain associations with an independent gene-family annotation from the Molecular Signatures Database (MSigDB) (38), which showed that GMs and SFs were mostly growth factors and cytokines, MRs were mostly cell surface differentiation markers and receptor tyrosine kinases, ICRS were mostly kinases, whilst transcription factors dominated the ICNRS class (Supplementary Figure S7). Each of the five previously considered gene classes (Supplementary Tables S2–6) were then mapped onto these signaling domains. Combining GM and SF into an extra-cellular (EC) domain class, as well as ICRS and ICNRS into an intra-cellular (IC) category, and further combining the EC class with the transmembrane (MR) class, we observed a striking difference between the patterns of enrichment of the various driver alterations in relation to whether they mapped to the IC or EC+MR classes (Figure 2). Specifically, in 9/10 cancer types, we observed that genes undergoing hypermethylation and underexpression were significantly (Fisher-test *P* < 0.05) more likely to map to the EC+MR signaling domain compared to the IC domain (Figure 2A). This pattern was generally stronger for hypomethylated and overexpressed genes with 10/10 cancer types exhibiting significance at *P* < 0.05 level (Figure 2B). In contrast, SCNAs revealed an exact opposite pattern, with deleted underexpressed genes mapping more likely to the IC domain in 9/10 cancer types (Figure 2C) and with amplified overexpressed genes doing so also in 9/10 cancer types (Figure 2D). Genetic mutations did not reveal a consistent pattern of differential enrichment among signaling domains (Figure 2E). Meta-analysis *P*-values confirmed that all of these associations were highly significant across cancer-types (Supplementary Table S8). To ensure that these results were not biased by different numbers of genes in each molecular alteration group, we repeated the analysis setting the number of genes in each group to be the same (the smallest number among all five gene groups), confirming that results are robust (Supplementary Figure S8 and Supplementary Table S9).

Although the previous results were obtained on sets of genes that exhibit differences between normal and cancer tissue, we asked if the enrichment of epigenetically altered genes within the EC+MR class is also true for epigenetically regulated tissue-specific genes in a given normal tissue-type. To assess this, we derived for each tissue-type a set of genes which were hypermethylated and underexpressed, or hypomethylated and overexpressed, in that tissue compared to the other nine tissue types considered here (‘Materials and Methods’ section). We observed that these tissue-specific DNAm and mRNA expression altered genes exhibited significant overlap with the previously derived cancer altered genes (Supplementary Figure S9) and that these tissue-specific genes were therefore also enriched among the EC+MR signaling domain class (Supplementary Figure S10 and Supplementary Table S10). This data is consistent with the view that tissue-specific genes are often differentially expressed in cancer and that this deregulation is associated with epigenetic alterations (41).

### Pan-cancer wide analysis identifies signaling pathways exhibiting differential signaling domain enrichment of epigenetic versus genetic alterations

**Figure 3.**
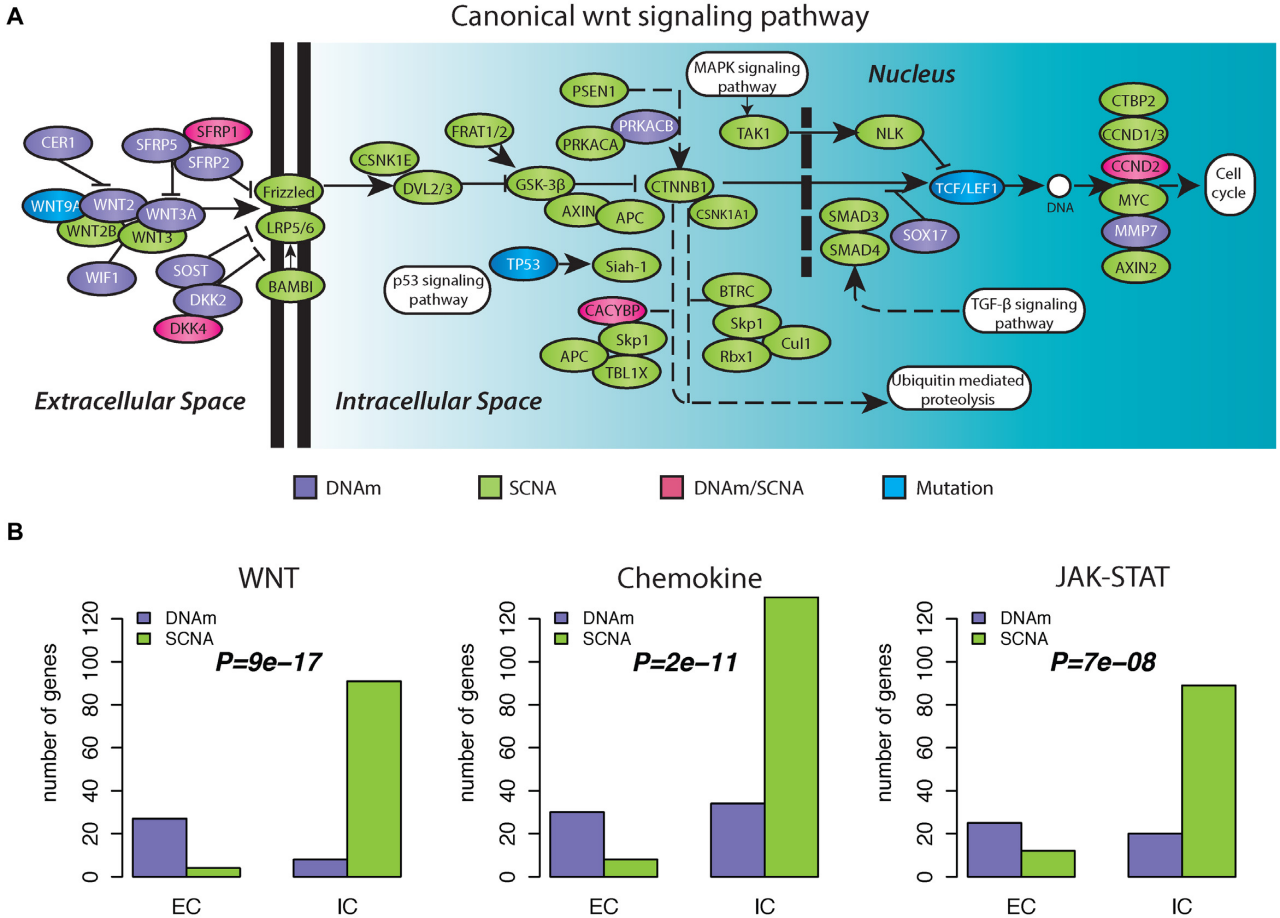
Differential signaling domain enrichment of epigenetic versus genetic alterations within the canonical WNT-signaling pathway. (**A**) Map of the canonical WNT-signaling pathway, with genes undergoing different types of alterations (indicated in different colors) across 10 cancer types. DNAm: a gene exhibits only functional epigenetic aberrations, whether it is hypermethylated or hypomethylated, in one or more cancer types. SCNA: a gene exhibits only SCN aberrations, whether it is amplified or deleted, in one or more cancer types. DNAm/SCNA: a gene exhibits both functional epigenetic aberrations and SCN aberrations in different cancer types. Mutation: a gene exhibits only mutations in one ore more cancer types. From left to right is the direction of cells from outside to inside. (**B**) Barplots comparing the cumulative sum of the alterations over all 10 cancer types in three signaling pathways, distributed according to different signaling domains (IC-intracellular and EC-extracellular) and alteration type (DNAm: functional epigenetic aberrations and SCNA: SCN aberrations). From left to right are canonical WNT signaling pathway as indicated in panel A, Chemokine signaling pathway and JAK-STAT signaling pathway. *P*-values were calculated using a one-tailed Fisher's exact test, testing the alternative hypothesis that there are proportionally more epigenetic alterations in the EC domain compared to SCNAs.

In order to identify specific signaling pathways which exhibit differential signaling domain enrichment between DNAm and SCNAs, we computed the number of genes undergoing functional DNAm or SCN alterations in each major signaling pathway domain and for all major signaling pathways (‘Materials and Methods’ section). For each cancer-type and signaling pathway we obtained a *P*-value to test for enrichment of functional DNAm alterations in the extracellular domain. In a meta-analysis over all 10 cancer types, specific signaling pathways emerged as exhibiting a consistent differential enrichment pattern across cancer-types (Table 2). Among the most highly ranked pathways, we found G-Protein Coupled Receptor (GPCR) signaling, immune system and chemokine signaling and JAK-STAT signaling (Table 2). WNT-signaling, a hotspot of age-associated differential DNAm in normal tissue (17), was also one of the highest ranked pathways, attaining significant *P*-values in 7/10 tumor types (Combined Fisher-test *P* < 0.0001) (Table 2). Focusing on the canonical WNT-signaling pathway, we confirmed a clear differential enrichment across signaling domains, with most of the epigenetic alterations occurring in the extra-cellular domain (Figure 3A). Aggregating numbers of alterations across all 10 cancer-types further confirmed a strong differential enrichment within the WNT-signalling pathway, the Chemokine signaling pathway and the JAK-STAT signaling pathway (Figure 3B). We note that many of the identified signaling pathways, including WNT-signaling, exhibited an enrichment toward functional DNAm alterations in the extracellular domain regardless of the genomic pattern of alteration (Supplementary Table S11). Importantly, we did not observe any signaling pathway to be significant if we tested for a reverse enrichment pattern, i.e. one with more functional DNAm alterations in the intra-cellular domain, either in comparison to genes undergoing functional SCNAs or not (data not shown), further supporting the view that cancer cells exhibit a preference for extracellular and transmembrane genes to undergo epigenetic deregulation.

**Table 2.** Top-ranked signaling pathways exhibiting a significant differential signaling domain distribution between functional DNA methylation and SCN alterations based on a meta-analysis over 10 cancer types

Signaling pathway	BLCA	BRCA	COAD	HNSC	KIRC	LIHC	LUAD	LUSC	THCA	UCEC	ALL
GPCR	0.103	0.024	0.116	4e-05	2e-04	0.022	0.031	0.002	1	0.116	7e-10
IMMUNE SYSTEM	0.006	1	0.017	0.117	0.001	0.118	8e-04	0.176	0.009	0.016	2e-08
WNT	0.061	0.016	0.006	0.015	0.008	0.011	0.008	0.035	0.1	1	8e-08
CHEMOKINE	0.05	0.005	0.611	0.001	9e-04	0.053	0.5	0.133	0.05	0.455	2e-06
HEMOSTASIS	0.045	4e-05	0.551	0.004	0.072	0.154	0.038	0.498	1	0.022	2e-06
FOCAL ADHESION	0.331	0.038	0.261	0.056	0.095	0.182	0.083	0.005	1	0.006	1e-04
JAK_STAT	0.133	0.011	0.035	0.119	0.017	0.111	0.22	0.192	0.1	1	5e-04
TOLL_LIKE RECEPTOR	1	0.059	1	0.048	0.005	0.083	0.045	0.091	1	0.029	0.001
LIPID METABOLISM	0.03	0.013	0.082	0.154	1	0.2	0.015	1	0.044	1	0.003
DIABETES	0.1	0.004	0.429	0.1	1	0.012	0.077	0.6	1	1	0.012
PDGF	1	0.032	0.029	0.045	1	0.091	0.308	0.333	1	0.031	0.014

*P*-values for each cancer type were calculated using a one-tailed Fisher's exact test. A meta-analysis *P*-value was calculated for each signaling pathway using Fisher's combined test.

**Figure 4.**
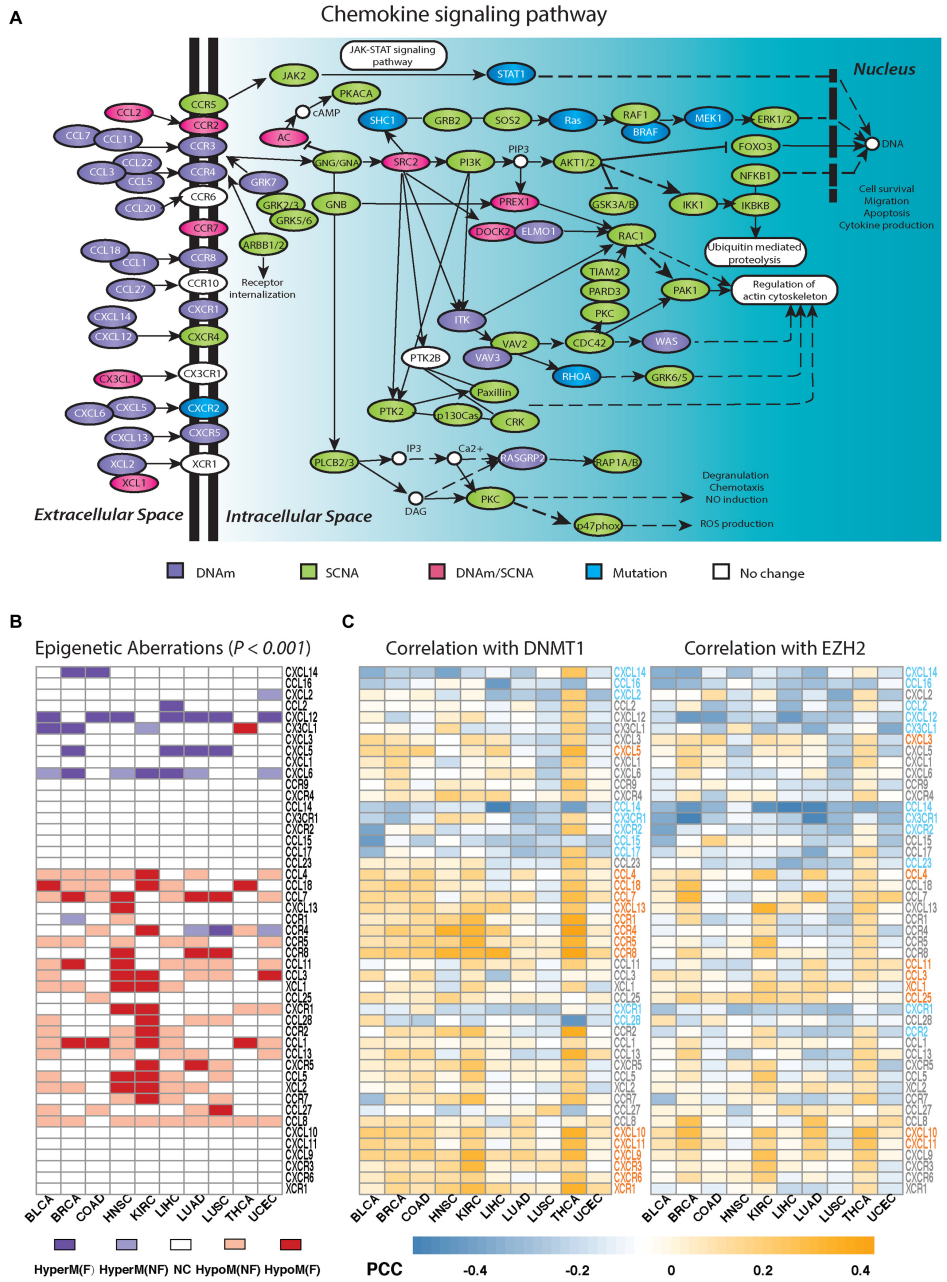
Differential signaling domain enrichment of epigenetic versus genetic alterations within the chemokine signaling pathway. (**A**) As Figure 3A, but now for the chemokine signaling pathway. (**B**) Heatmap depicts pattern of epigenetic deregulation in cancer for chemokine and chemokine receptors across 10 cancer types. HyperM(F): gene exhibits simultaneous hypermethylation and underexpression in cancer. HyperM(NF): gene is hypermethylated but not underexpressed. HypoM(F): gene exhibits significant hypomethylation and overexpression. HypoM(NF): gene is hypomethylated but not overexpressed. NC: gene shows no significant DNAm change in cancer. (**C**) Heatmaps depict Pearson correlation coefficients (PCCs) of gene expression for each chemokine and chemokine receptor with DNMT1 or EZH2, as computed using only tumor samples from each tumor type. Gene symbols are marked in different colors, indicating significant and directionally consistent correlation patterns across different cancer types. Gray: a gene shows no significantly consistent correlation pattern. Orange: a gene shows significantly consistent positive correlation with DNMT1 or EZH2. Blue: a gene shows significantly consistent negative correlation with DNMT1 or EZH2.

Besides WNT-signaling, chemokine signaling is also thought to play a major role in cancer progression, by upsetting the balance between a favorable Th1-type and an adverse Th2-type immune response (42–44). Mapping the functional alterations across cancer-types onto a global chemokine signaling pathway confirmed a striking differential enrichment, with functional epigenetic deregulation happening mostly in the extracellular domain (Figure 4A). We verified for individual chemokines and chemokine receptors, that patterns of epigenetic deregulation were highly consistent between cancer types (Figure 4B, Empirical *P* < 0.001), demonstrating that these patterns of deregulation transcend the tissue/cancer type.

Two important epigenetic enzymes which are universally overexpressed in cancer (45), and which are known to influence DNAm levels are DNMT1 and EZH2 (46). Given their role in suppressing specific Th1-type chemokines in ovarian cancer (47), we asked if DNAm patterns of epigenetically deregulated genes in the chemokine signaling pathway were significantly correlated (or anti-correlated) with expression of either DNMT1 or EZH2, and how this varied across cancer-types. Interestingly, this revealed that some chemokines and chemokine receptors were generally always either correlated or anti-correlated with expression of these two enzymes across cancer-types (Figure 4C), pointing toward universal patterns of co-expression with key epigenetic enzymes. Interestingly, chemokines or chemokine receptors exhibiting consistent hypermethylation and underexpression in cancer, exhibited expression patterns across tumors that were more likely to be consistently negatively correlated with expression of EZH2 or DNMT1 (or both) (Figure 4B and C). In contrast, for those exhibiting consistent hypomethylation/overexpression in cancer, their expression across tumors is more likely to be consistently positively correlated with EZH2 or DNMT1 (or both). We note that for many genes that showed a significant and consistent correlation with expression of EZH2/DNMT1, about half of these (e.g. CCL14, CCL15, CXCL9, CXCL10, CXCL11, CXCR3, CXCR6) did not have any 450k probe mapping to their TSS200, first exon or TSS1500 regions, not allowing DNAm changes around the promoter to be assessed.

## DISCUSSION

Here we have conducted a systems-level comparative analysis of functional DNAm and SCN alterations, including mutations, in cancer. Our three key findings, (i) that functional DNAm alterations exhibit a significantly lower connectivity compared to functional SCNAs and mutations, (ii) that functional DNAm alterations tend to target genes in the extracellular and transmembrane domains and (iii) that there exist specific signaling pathways (e.g. chemokine and WNT signaling) which exhibit such preferential epigenetic deregulation in the extracellular domain independently of cancer-type, shed novel insight into the potentially distinctive role of these alteration types in cancer.

Previous studies have shown that DNAm changes in cancer and aging are enriched for bivalently and PRC2 marked genes, which in turn are highly enriched for developmental transcription factors (TFs) (48–52). These TFs occupy peripheral positions in a PPI network like the one considered here, which does not include explicit regulatory protein–DNA interactions. However, that TFs map to the periphery of our PPI network, does not imply that functional DNAm alterations in cancer would also occupy peripheral positions, because most of the PRC2 marked TFs undergoing promoter DNAm in cancer are not altered at the expression level (as they are generally not expressed in the normal tissue to begin with) (53). Thus, it is not a foregone conclusion that the subset of genes undergoing epigenetic deregulation in cancer would necessarily mark nodes of low connectivity. Indeed, our second key finding indicates that the lower connectivity of functional DNAm alterations in cancer, is driven mainly by genes encoding growth modulators and secreted factors.

Interestingly, a similar enrichment for genes in the extracellular domain, was also observed for tissue-specific genes for which their tissue-specific expression level is strongly associated with the degree of DNAm at their promoter. We observed that this similar enrichment can be explained by the fact that there was considerable overlap between the epigenetically regulated tissue-specific genes and those genes undergoing simultaneous differential methylation and expression in cancer, consistent with previous findings (41). Indeed, one of the main cancer hallmarks is a lack of differentiation, so it should not be surprising that tissue-specific genes are preferentially altered in cancer. Hence, our observation that functional DNAm alterations in cancer are enriched within the extracellular domain can be partially explained by the corresponding enrichment of tissue-specific genes.

The enrichment of functional DNAm alterations within the extracellular domain, in contrast to SCNAs (which were over-represented in the intracellular space) and to genetic mutations (which did not exhibit any differential enrichment pattern), was highly consistent across cancer-types, attesting to its biological significance. Our third key finding showed that there exist specific signaling pathways which are more prone to epigenetic deregulation in their extracellular signaling domain, irrespective of cancer-type. This included two signaling pathways of critical importance in carcinogenesis: WNT- and chemokine signaling (44,54).

We stress that our observation that these specific pathways are prone to epigenetic deregulation irrespective of cancer-type, is, to the best of our knowledge, an entirely novel insight. It is important, because evidence is mounting that epigenetic alterations, or genetic modulation of epigenetic regulators, also contribute to carcinogenesis (1,6,55,56). Like genetic mutations and somatic copy-number changes, epigenetic alterations also accrue in normal cells as a function of age and as a function of exposure to cancer risk factors. However, because the epigenome is more easily modulated than the genome, the epigenome is the prime candidate to mediate the effects of environmental exposures (57). These exposures are, by definition, cell-extrinsic, mediated by alterations in the environmental niche, in which adult stem-cells of the underlying tissue reside. It is therefore plausible that cellular adaptation to extra-cellular stresses would involve a mechanism that targets the proteins that mediate the extra-cellular signals. Although signal transduction is a complex biological process, involving proteins at every layer of the signaling domain hierarchy, it can be argued that the most direct means to adapt to specific extra-cellular signals is through modulation of extracellular factors and to a less degree by transmembrane receptors. Indeed, it has already been demonstrated that expression variability, as assessed across a large number of different normal tissue types, is maximal for genes whose main cellular localization at the protein level is in the extra-cellular domain (37). Many expression markers of specific cell-types also map to the cell-surface. As we have shown here, the subset of genes in the extracellular and transmembrane domains which get functionally altered in cancer, appear to do so preferentially through alterations in DNAm. Thus, epigenetic deregulation of extracellular signaling domain genes in cancer may reflect adaptation of cancer cells to a selection process driven by specific environmental stresses. This interpretation is strongly supported in the case of the WNT-signaling pathway, as many previous studies have demonstrated that WNT activity in epithelial stem-cells is controlled by cell-extrinsic factors and that modulation of WNT activity affects sensitivity of cells to DNA damage, thus linking epigenetic deregulation which may happen early in carcinogenesis to an increased predisposition to acquire genetic alterations (58–61).

The role of the immune response in controlling the risk of distant metastasis and hence of clinical outcome in cancer is well established (62,63). A long-standing observation, supported by analysis of gene expression data, is that a T-helper-1 type immune response is generally associated with a favorable prognosis, in contrast to an opposing macrophage polarization program which promotes an unfavorable T-helper-2 type response (42,43). The important role of epigenetics in shaping the type of immune response in the tumor microenvironment was recently demonstrated by Peng *et al.* (47), where it was shown how epigenetic mediated silencing of specific Th1-type chemokines could promote ovarian cancer progression and lessen the therapeutic efficacy of programmed death-ligand 1 checkpoint blockade. Unfortunately, the specific chemokine ligands considered by Peng *et al.* (e.g. *CXCL9* and*CXCL10*) do not have 450k probes mapping to their promoters and our study also excluded ovarian cancer due to the lack of an appropriate normal reference. Nevertheless, our pan-cancer wide analysis of the chemokine signaling pathway revealed a striking pattern of epigenetic deregulation, with several chemokines/chemokine receptors exhibiting consistent hypermethylation and underexpression in cancer, also exhibiting expression patterns (across tumors) that correlated negatively with either DNMT1 or EZH2 (or both). It is very likely that these chemokine genes play a tumor suppressor role in cancer, and their consistent negative correlation with expression of epigenetic enzymes such as DNMT1 or EZH2, suggest that their underexpression may be under epigenetic control. For instance, our analysis identified promoter hypermethylation and underexpression of ligand CXCL12 in six cancer types, and previous studies have reported epigenetically induced silencing of this gene in breast cancer (64), colon cancer (65) and non-small cell lung cancers (66). Hypermethylation of CXCL12 in non-small cell lung cancer has also been reported to be a poor prognostic marker (66). Another interesting chemokine ligand is CXCL14, which we observed to be hypermethylated and underexpressed in two cancer types (breast and colon), but which exhibited a distinctive anti-correlative expression pattern with EZH2/DNMT1 in most cancer types. Supporting this, epigenetic silencing of CXCL14 has been found to promote progression of breast (67), colorectal (68) as well as gastric cancer (69).

In stark contrast to DNAm, functional SCNAs appear to preferentially target genes in the intra-cellular domain, affecting central processes such as the cell-cycle. Kinases, phosphatases and other intra-cellular receptor substrates are characterized by a significantly higher level of signaling promiscuity and centrality. Disruption of genes in this signaling domain may contribute to increased cell-proliferation, but largely also toward an increased cellular resistance and robustness (70,71).

In summary, this work exposes a deep subtle difference between functional epigenetic and genetic alterations in cancer, suggesting that these molecular alterations may contribute in distinct ways to the carcinogenic process.
